# Corticosterone Attenuates Reward-Seeking Behavior and Increases Anxiety via D2 Receptor Signaling in Ventral Tegmental Area Dopamine Neurons

**DOI:** 10.1523/JNEUROSCI.2533-20.2020

**Published:** 2021-02-17

**Authors:** Beibei Peng, Qikuan Xu, Jing Liu, Sophie Guo, Stephanie L. Borgland, Shuai Liu

**Affiliations:** ^1^Key Laboratory of Brain Functional Genomics (MOE&STCSM), Affiliated Mental Health Center (ECNU), School of Psychology and Cognitive Science, East China Normal University, Shanghai, 200062, China; ^2^Shanghai Changning Mental Health Center, Shanghai, 200335, China; ^3^NYU-ECNU Institute of Brain and Cognitive Science at NYU Shanghai, Shanghai, 200062, China; ^4^Department of Physiology & Pharmacology, Hotchkiss Brain Institute, Cumming School of medicine, University of Calgary, Calgary, Alberta T2N 4N1, Canada

**Keywords:** anxiety, corticosterone, D2 receptor, dopamine, reward, ventral tegmental area

## Abstract

Corticosteroids (CORT) have been widely used in anti-inflammatory medication. Chronic CORT treatment can cause mesocorticolimbic system dysfunctions, which are known to play a key role for the development of psychiatric disorders. The VTA is a critical site in the mesocorticolimbic pathway and is responsible for motivation and reward-seeking behaviors. However, the mechanism by which chronic CORT alters VTA dopamine neuronal activity is largely unknown. We treated periadolescent male mice with vehicle, 1 d, or 7 d CORT in the drinking water, examined behavioral impacts with light/dark box, elevated plus maze, operant chamber, and open field tests, measured the effects of CORT on VTA dopamine neuronal activity using patch-clamp electrophysiology and dopamine concentration using fast-scan cyclic voltammetry, and tested the effects of dopamine D2 receptor (D2R) blockade by intra-VTA infusion of a D2R antagonist. CORT treatment induced anxiety-like behavior as well as decreased food-seeking behaviors. We show that chronic CORT treatment decreased excitability and excitatory synaptic transmission onto VTA dopamine neurons. Furthermore, chronic CORT increased somatodendritic dopamine concentration. The D2R antagonist sulpiride restored decreased excitatory transmission and excitability of VTA dopamine neurons. Furthermore, sulpiride decreased anxiety-like behavior and rescued food-seeking behavior in mice with chronic CORT exposure. Together, 7 d CORT treatment induces anxiety-like behavior and impairs food-seeking in a mildly aversive environment. D2R signaling in the VTA might be a potential target to ameliorate chronic CORT-induced anxiety and reward-seeking deficits.

**SIGNIFICANCE STATEMENT** With widespread anti-inflammatory effects throughout the body, corticosteroids (CORT) have been used in a variety of therapeutic conditions. However, long-term CORT treatment causes cognitive impairments and neuropsychiatric disorders. The impact of chronic CORT on the mesolimbic system has not been elucidated. Here, we demonstrate that 7 d CORT treatment increases anxiety-like behavior and attenuates food-seeking behavior in a mildly aversive environment. By elevating local dopamine concentration in the VTA, a region important for driving motivated behavior, CORT treatment suppresses excitability and synaptic transmission onto VTA dopamine neurons. Intriguingly, blockade of D2 receptor signaling in the VTA restores neuronal excitability and food-seeking and alleviates anxiety-like behaviors. Our findings provide a potential therapeutic target for CORT-induced reward deficits.

## Introduction

Corticosteroids (CORT) have been used for a variety of conditions, such as pneumonia, asthma, severe allergies, and arthritis, because of its widespread anti-inflammatory effects (Rhen and Cidlowski, 2005). However, long-term exposure to CORT medications may cause side effects, including cognitive impairments and neuropsychiatric disorders (Sonino and Fava, 2001; Coluccia et al., 2008; Pivonello et al., 2015; Yasir and Sonthalia, 2019). While the physiological effects of acute CORT in the brain are well documented (Mitra and Sapolsky, 2008; Kim et al., 2014; Myers et al., 2014; Joels, 2018), the impact of longer-term CORT treatment, consistent with systemic anti-inflammatory treatment, on the mesolimbic system has not been elucidated.

VTA dopamine neurons play an important role in learning the incentive value of stimuli or actions to guide motivated behavior and thus play key roles in addiction and mood disorders. These neurons are highly sensitive to corticotropin releasing factor and stress (for review, see Polter and Kauer, 2014; Hollon et al., 2015), yet less is known about how they respond to glucocorticoids. CORT activates glucocorticoid receptors (GRs) or mineralocorticoid receptors (MRs), and these receptors are subsequently translocated to the nucleus and act as transcription factors (McEwen et al., 1986). However, rapid nongenomic cellular mechanisms have been observed on activation of GRs (Tasker and Herman, 2011).

Several lines of evidence indicate that the midbrain dopamine system could be implicated in impairments associated with chronic CORT treatment. Both MRs and GRs are expressed in a subset of VTA dopamine neurons (Harfstrand et al., 1986; Ronken et al., 1994; Hensleigh and Pritchard, 2013). Activation of GRs by either *in vivo* injection or acute incubation of VTA slices with dexamethasone potentiates synaptic strength onto dopamine neurons (Daftary et al., 2009). Furthermore, stress-induced plasticity in the VTA is blocked by administration of a GR antagonist (Saal et al., 2003). Acute CORT application potentiated NMDA currents in VTA slices (Cho and Little, 1999) and NMDA neurotoxicity of VTA neurons in organotypic VTA and NAc cocultures (Berry et al., 2016). Because major depressive disorder and reduced motivation are frequently reported in patients with CORT treatment-induced Cushing syndrome (Wagenmakers et al., 2012; Pivonello et al., 2015), chronic CORT treatment may dysregulate dopamine signaling to influence behavior. However, little is known about how chronic CORT treatment influences synaptic transmission in the VTA. In the present study, we tested the hypothesis that chronic CORT exposure impacts VTA neurophysiology and reward-seeking behaviors using an oral CORT treatment mouse model (Gourley et al., 2008; Kinlein et al., 2017).

## Materials and Methods

### 

#### Animals and CORT treatment

All protocols were in accordance with the ethical guidelines established by the Canadian Council for Animal Care and were approved by the University of Calgary Animal Care Committee and by the East China Normal University Animal Care Committee. P21 male C57BL/6 mice obtained from Charles River Laboratories or The Jackson Laboratory were housed in a 12 h light/12 h dark cycle in a temperature- and humidity-controlled environment with food and water freely available. All efforts were made to minimize animal suffering and reduce the number of animals used. Oral CORT treatment was conducted by replacing drinking water with either 100 µg/ml CORT dissolved in ethanol (1% ethanol final concentration) or vehicle (1% ethanol solution) (Kinlein et al., 2017; Moda-Sava et al., 2019). Body weight, food consumption, and water intake were measured. To measure CORT level in mice, blood was collected at 3-5 h after light on and clotted at room temperature for ∼30 min followed by centrifugation (955 × *g* for 30 min at 4°C). The serum was stored at −80°C before assaying. Serum was diluted 1:500 for corticosterone ELISA (Cayman Chemical, #501320) following the assay instruction. Glucose tolerance test was performed using glucose meter (Roche Diagnostic). After overnight fasting, animals were weighed and basal glucose was measured. D-glucose was then injected (2 g/kg, 0.9% saline, i.p.), and tail blood was collected at 10, 20, 30, 45, 60, 90, 120, and 180 min and measured with a glucometer.

#### Electrophysiology and electrochemistry

Slice electrophysiology experiments were performed from 4- to 5-week-old male C57BL/6 mice. Mice were anesthetized with either isoflurane or 0.6% pentobarbital sodium, decapitated, and brains were extracted. VTA slices (250 μm) were prepared with an ice-cold sucrose solution containing (in mm): 87 NaCl, 2.5 KCl, 1.25 NaH_2_PO_4_, 25 NaHCO_3_, 7 MgCl_2_, 0.5 CaCl_2_, 75 sucrose, using a vibratome (Leica Microsystems). Slices were placed in a holding chamber and allowed to recover for at least 1 h before moving to the recording chamber and superfused with aCSF solution containing the following (in mm): 126 NaCl, 1.6 KCl, 1.1 NaH_2_PO_4_, 1.4 MgCl_2_, 2.4 CaCl_2_, 26 NaHCO_3_ and 11 glucose, saturated with 95% O_2_/5% CO_2_ at 32°C. Cells were visualized using infrared differential interference contrast video microscopy.

**Figure 1.**
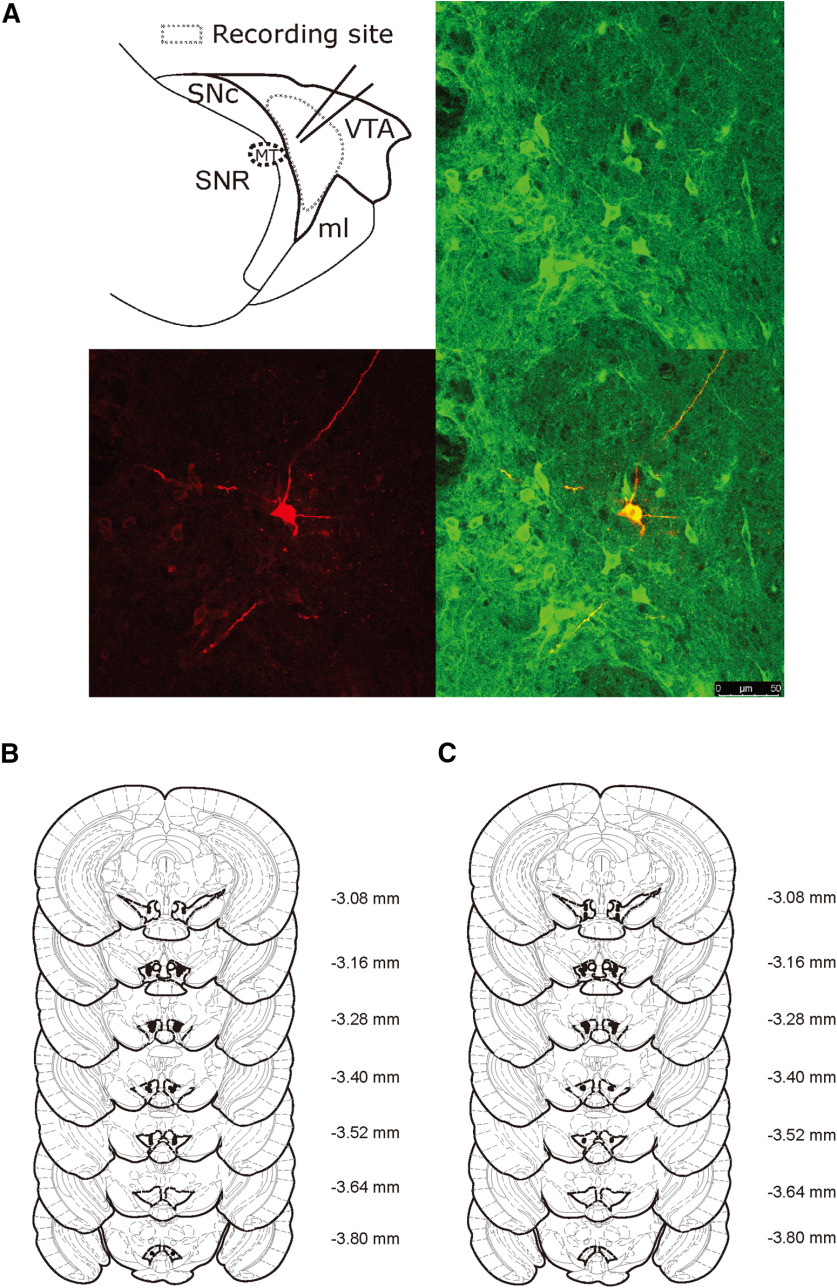
Schematic recording sites and reconstructed bilateral injection sites in the VTA. ***A***, Schematic recording sites (top left). Example confocal image of biocytin-labeled TH-positive neuron. *Post hoc* TH immunofluorescence was identified in the VTA (top right). Biocytin was administered via the patch pipette during the electrophysiological recording (bottom left). Merged image of a biocytin-labeled TH-positive neuron (bottom right). Scale bar, 50 µm. ***B***, Injection sites in the reward-seeking behavior test and (***C***) injection sites in the OF, EPM, and PR tests are shown in coronal sections. Distance from bregma is shown to the right of each section (in millimeters).

Whole-cell patch-clamp recordings were made using a MultiClamp 700B amplifier (Molecular Devices). Electrodes (3-5 mΩ) contained the following (in mm): 117 cesium methanesulfonate, 20 HEPES, 0.4 EGTA, 2.8 NaCl, 5 TEA-Cl, 2.5 Mg-ATP, and 0.25 Na-GTP (pH 7.2-7.3, 270-280 mOsm). Biocytin (0.2%) was added to the internal solution before every experiment. Series resistance (10-25 mΩ) and input resistance were monitored online with a 10 mV depolarizing step (400 ms) given before every afferent stimulus. Dopamine neurons were identified by the presence of a hyperpolarizing cation current, a predictor of lateral VTA dopamine neurons medial to the medial terminals of the optic nucleus in mice (Wanat et al., 2008) and were further confirmed by *post hoc* staining for the rate-limiting enzyme, TH (Fig. 1*A*).

A bipolar stimulating electrode was placed 100-300 μm rostral to the recording electrode to stimulate excitatory afferents at 0.1 Hz. AMPAR-mediated EPSCs were recorded in cells voltage-clamped at −60 mV in the presence of picrotoxin (100 μm) to block GABA_A_ receptor-mediated IPSCs. EPSCs were filtered at 2 kHz, digitized at 10 kHz, and collected using pClamp 10 (Molecular Devices). Currents traces were constructed by averaging 10 consecutive EPSCs. Paired pulses were evoked with a 50 ms interstimulus interval. To calculate the AMPAR/NMDAR ratio, an average of 12 EPSCs at 40 mV was computed before and after application of the NMDAR blocker AP5 (50 μm) for 5 min. NMDAR responses were calculated by subtracting the average response in the presence of AP5 (AMPAR only) from that seen in its absence; the peak of the AMPAR EPSC was divided by the peak of the NMDAR EPSC to yield an AMPAR/NMDAR ratio.

To test excitatory and inhibitory transmission, and calculate their ratio, we recorded mEPSCs and mIPSCs from same neuron. Inhibitory and excitatory current reversal potentials were first tested and calculated from *I-V* curve, respectively. mEPSCs were recorded in cells voltage-clamped at −68 mV (inhibitory reversal potential) in TTX (500 nm). mIPSCs were voltage clamped at 10 mV (excitatory reversal potential) in TTX (500 nm). mIPSCs and mEPSCs were analyzed using Mini60 Mini Analysis Program (Synaptosoft). Detection criteria were set at >12 pA (at least 2× rms noise), <1.75 ms rise time and <4 ms decay time for AMPAR mEPSCs, and for mIPSCs >15 pA (at least 2× rms noise), <4 ms rise time and <10 ms decay time to account for the longer decay kinetics of GABA_A_ receptors (Godfrey and Borgland, 2020).

To investigate neuronal excitability, glass electrodes (3-5 mΩ) contained the following (in mm): 140K D-gluconate, 5 KCl, 10 HEPES, 0.2 EGTA, 2 NaCl, 2.5 Mg-ATP, and 0.25 Na-GTP (pH 7.2-7.3, 270-280 mOsm). Neurons were held at −60 mV and depolarizing current steps (400 ms, from 0 to 350 mV in 50 mV steps) were applied in the absence of transmitter receptor blockers. The slope of the number of spikes per depolarizing step was used as a measure of excitability.

Evoked extracellular dopamine ([DA]_o_) was measured using fast-scan cyclic voltammetry with carbon-fiber microelectrodes as previously reported (Mebel et al., 2012). Briefly, carbon fibers (7 μm diameter; Goodfellow) were pulled in glass electrodes and cut to a final exposed length of ∼150 μm. Triangular waveforms (holding at −0.4 V) at 10 Hz (−0.4 to 1.0 V vs Ag/AgCl at 400 V/s scan rate) were used. Electrical stimulation (40 Hz, 20 pulses) was applied with a bipolar stimulating electrode positioned flush with the tissue for local surface stimulation. The voltammetric electrode was positioned between the tips with the aid of a binocular microscope, then lowered 50-100 μm into the tissue. Dopamine was identified by characteristic oxidation and reduction peak potentials (600 and −200 mV vs Ag/AgCl). To determine the time course of dopamine, the current at the peak oxidation (600 mV) was plotted against time. Calibration solutions were made from stock solutions in 0.1 m HClO_4_ immediately before use.

#### Behavioral tests

##### Reward-seeking behavior test

The reward-seeking behavior test was modified from light/dark box test (Teegarden and Bale, 2007), whereby mice were required to overcome their innate aversion to open light spaces to seek high-fat (HF) food. The apparatus was an acrylic box (45 cm length × 27 cm width × 27 cm height) divided into light and dark chambers. The light chamber (27 cm length × 27 cm width × 27 cm height) was of white acrylic and was connected via an opening (7.5 × 7.5 cm) at floor level to the black acrylic chamber (18 cm length × 27 cm width × 27 cm height). A pellet of HF food (Research Diets, d12492) was placed in the center of the light chamber, and the area around food (9 cm × 9 cm) was defined as food zone. Mice were pre-exposed to HF food briefly 2 d before the test to avoid neophobia. Mice were habituated to the test room before the test. A lamp was placed 2.5 m above the test chamber creating 92.6-108 lux of light. Mice were placed in the light chamber facing the opening into the dark chamber, and Any-Maze software was used to record and track the mice during a 10 min trial. The apparatus was cleaned between each subject.

##### Elevated plus maze (EPM)

The acrylic maze consists of 2 open arms and 2 closed arms (30 cm in length and 5 cm in width, 15 cm wall for closed arm). Distance traveled and time spent in each arm during 10 min tests were recorded and analyzed by Any-Maze software. The apparatus was cleaned between each subject.

##### Open field test (OF)

Tru scan system with an arena of 26 cm (length) × 26 cm (width) was used in the test. Mice were placed in the center of the arena. Distance traveled was recorded and analyzed automatically by the system during a 10 min trial. The apparatus was cleaned between each subject.

##### Progressive ratio test (PR)

Bussey-Saksida touchscreen chamber (Campden Instruments) was used in the PR test. During the training phase, mice were habituated to the chambers for 20 min with 200 μl milkshake (Yazoo Strawberry UHT milkshake; Friesland Campina) delivered to the magazine on the first day. After habituation, mice were trained to perform the specific operant response (fixed ratio [FR] training, FR1, FR3, FR5) to get a reward (20 µl milkshake) over several sessions (1 or 2 sessions per day). Criterion of a successful session was defined as completion of 30 trials in 60 min. FR5 was conducted three consecutive sessions to ensure animals developed high selectivity for the target-illuminated screen location. Following FR5, mice progressed to three consecutive PR schedule sessions, in which a reward was delivered with a progressively increasing operant response requirement (i.e., 1, 5, 9, 13, etc.). During the training phase, mice were food-restricted (∼85% of their daily food intake). After 7 d CORT treatment, three consecutive PR schedule tests were conducted. Task performance was evaluated by breakpoint, defined as the number of target responses emitted by an animal in the last successfully completed trial of a session.

##### Food preference test

Mice were tested for HF food preference using a two-bowl choice procedure. Each animal was housed individually during the 7 d test period. Animals were given two bowls, containing HF food and standard chow, respectively. Water was provided *ad libitum*. Every 24 h, the amounts of HF food and lab chow consumed were recorded. To prevent potential location preference, the positions of the bowls were changed every 24 h. The preference for the HF food was determined as the percentage of HF food ingested relative to the total food intake.

#### Intra-VTA cannulations and microinjections

Mice (4-5 weeks old) were anesthetized and placed in a stereotaxic frame (Kopf). Mice were implanted with bilateral guide cannulas (26 gauge; RWD 62064) into the VTA (anteroposterior, −3.2 mm; mediolateral, ± 0.5 mm; dorsoventral, −4.6 mm). For micro-infusions, mice had two habituation sessions, by performing mock perfusions with a microinjector cut above the length of the cannula. On test days, micro-infusions were conducted by using 32-gauge microinjectors (RWD 62264) that protruded 0.2 mm below the base of the guide cannula to a final dorsoventral coordinate of −4.8 mm. Vehicle (aCSF) or D2 receptor (D2R) antagonist sulpiride (0.15 µg/side) was infused bilaterally into the VTA (0.2 μl at 0.1 μl/min), and microinjectors were left in place for 3 min after the injection. Mice were then returned to their home cage for 15 min before the behavioral assay. Injection placements were checked after the test (Fig. 1*B*,*C*).

#### Drugs

Corticosterone, picrotoxin, and ketoprofen were purchased from Cayman Chemical. FITC-AffiniPure Donkey Anti-Mouse IgG (H + L) and AlexaFluor-594-IgG Fraction Monoclonal Mouse Anti-Biotin were purchased from Jackson ImmunoResearch Laboratories. TTX was purchased from the Research Institute of the Aquatic Products of Hebei. All other regents and drugs were purchased from Sigma Millipore, Tocris Bioscience, or BBI Life Sciences.

#### Data analysis

All values are expressed as mean ± SEM in addition to individual values. Statistical significance was assessed by using two-tailed Student's *t* tests. Two-way ANOVA was used for multiple group comparisons unless otherwise indicated. *N* refers to the number of cells recorded from *n* mice. Data met the assumptions of equal variances and normality unless otherwise indicated. Excel (Microsoft) and Prism (GraphPad Prism 7 Software) were used to perform data processing and statistical analysis.

## Results

### Impaired food-seeking and elevated anxiety in 7 d CORT-treated mice

**Figure 2.**
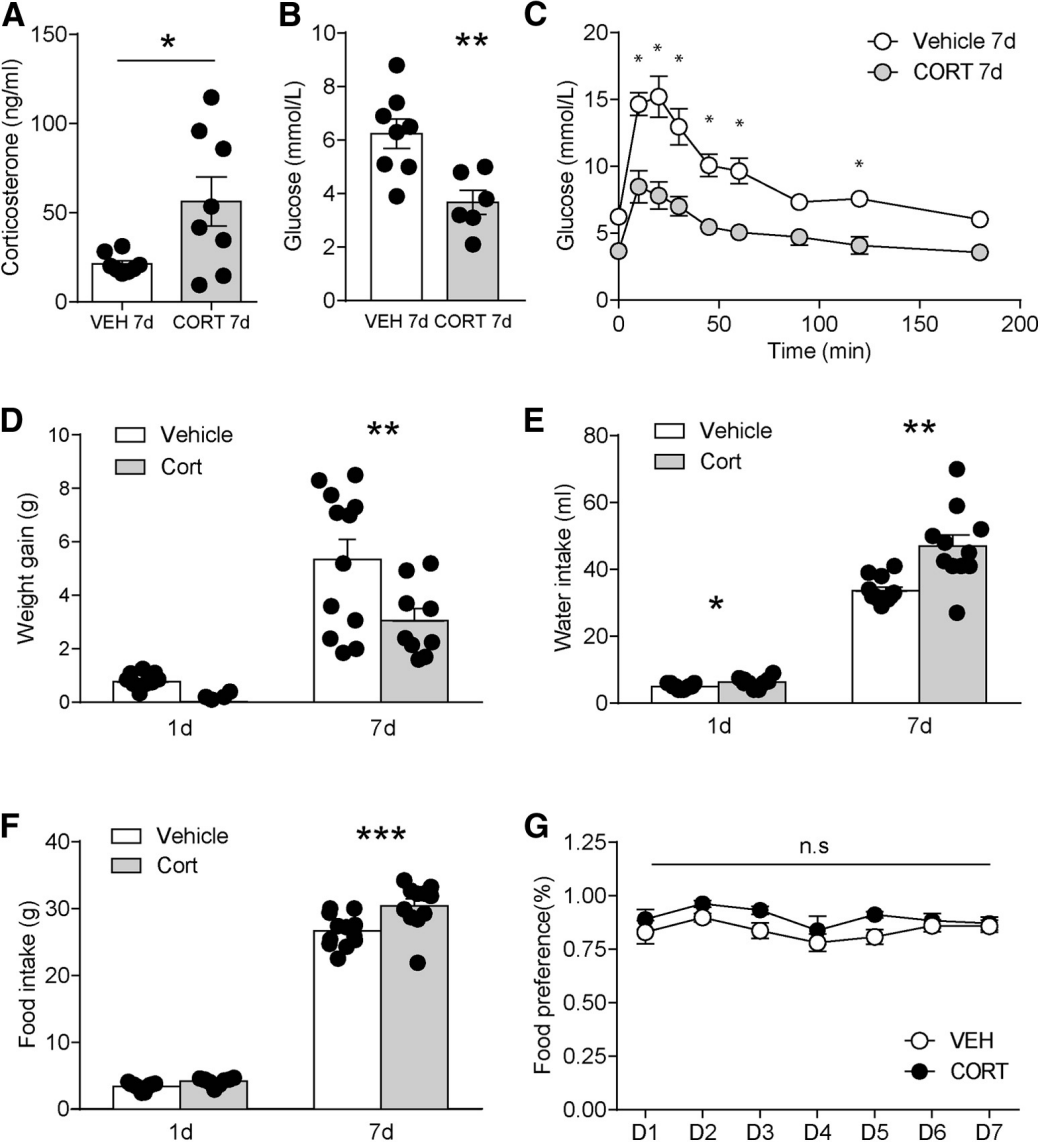
Metabolic changes in CORT-treated mice. ***A***, Serum corticosterone level. ***B***, Basal glucose level. ***C***, Glucose tolerance curve. ***D***, Weight gain. ***E***, Water intake. ***F***, Food intake. ***G***, Food preference for HF diet compared with chow during the 7 d vehicle or CORT treatment. Error bars indicate SEM. *p < 0.05. **p < 0.01. ***p < 0.001.

Chronic CORT treatment can induce metabolic dysfunction (Karatsoreos et al., 2010). Seven day CORT treatment significantly increased serum CORT level (vehicle: 21 ± 2.0, *n* = 8; CORT: 56 ± 14, *n* = 8; *t*_(14)_ = 2.5, *p* = 0.024; Fig. 2*A*). Baseline glucose level was significantly reduced in CORT mice after overnight fasting (vehicle: 6.2 ± 0.5, *n* = 8; CORT: 3.7 ± 0.4, *n* = 6; *t*_(12)_ = 3.5, *p* = 0.0048; Fig. 2*B*) with impaired glucose tolerance (*F*_(8,96)_ = 3.2, *p* = 0.0027; Fig. 2*C*). Body weight gain was significantly decreased in 7 d CORT mice compared with vehicle mice (vehicle: 5.3 ± 0.7 g, *n* = 12; CORT: 3.0 ± 0.4 g, *n* = 9; *t*_(19)_ = 2.4, *p* = 0.027; Fig. 2*D*), while water and food intake increased (water: vehicle: 34 ± 1 g, *n* = 12; CORT: 47 ± 3 g, *n* = 11; *t*_(21)_ = 3.9, *p* = 0.0008; food: vehicle: 27 ± 0.7 g, *n* = 12; CORT: 30 ± 1 g, *n* = 11; *t*_(21)_ = 3.1, *p* = 0.0055; Fig. 2*E*,*F*). Mice acutely exposed to CORT (1 d) had increased water intake (vehicle: 5 ± 0.2 g, *n* = 11; CORT: 6 ± 0.5 g, *n* = 10; water intake × CORT interaction: *F*_(1,40)_ = 11.16, *p* = 0.0018), while weight gain and food intake were not significantly different in 1 d CORT mice. However, CORT treatment did not change preference for HF food when both chow and HF food were provided in home cage (*F*_(6,84)_ = 0.49, *p* = 0.82; Fig. 2*G*), suggesting reward sensitivity per se was not altered. Together, these results suggest 7 d CORT has significant metabolic effects on mice.

**Figure 3.**
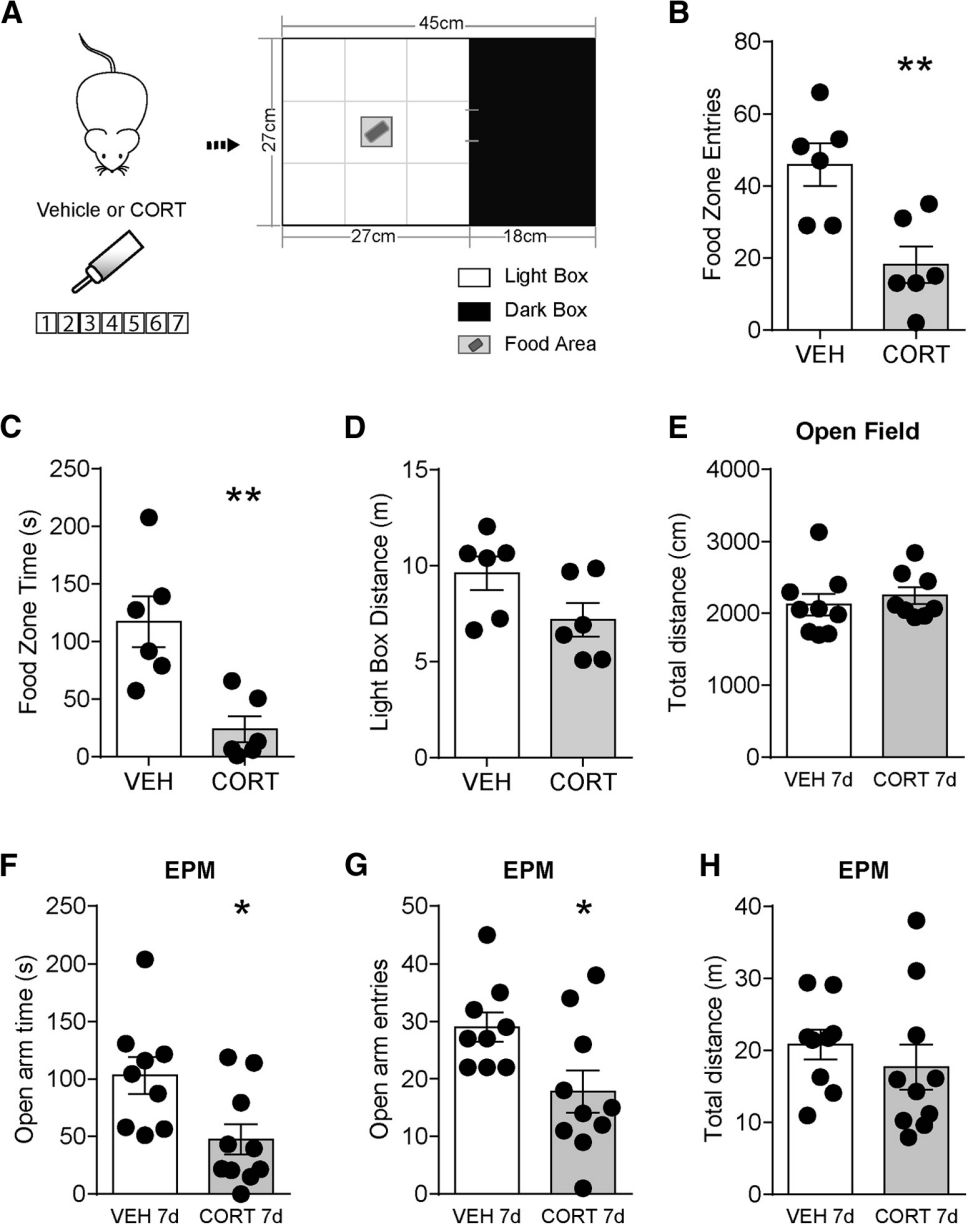
Seven day CORT treatment impairs reward-seeking behaviors and increases anxiety-like behavior. ***A***, Reward-seeking behavior test paradigm with HF food in the food zone. ***B***, Food zone entries, (***C***) food zone time, and (***D***) light box distance in vehicle or CORT mice. ***E***, Total distance in the OF test. ***F***, Open arm time, (***G***) open arm entries, and (***H***) total distance in the EPM test. Error bars indicate SEM. Not significant, *p* > 0.05. *p < 0.05. **p < 0.01.

To test whether chronic CORT treatment influences food-seeking, we used a modified light dark box, whereby mice must enter the open light field to explore food (Fig. 3*A*) (Teegarden and Bale, 2007; Liu et al., 2016). Seven day CORT treatment significantly decreased food zone entries (vehicle: 46 ± 6, *n* = 6; CORT: 18 ± 5, *n* = 6; *t*_(10)_ = 3.5, *p* = 0.0053; Fig. 3*B*) and food zone time (vehicle: 117 ± 22 s, *n* = 6; CORT: 24 ± 11 s, *n* = 6; *t*_(10)_ = 3.8, *p* = 0.0036; Fig. 3*C*), with no change in light box distance traveled (vehicle: 10 ± 0.9 m, *n* = 6; CORT: 7 ± 0.9 m, *n* = 6; *t*_(10)_ = 1.9, *p* = 0.079; Fig. 3*D*). In addition, CORT treatment did not change total distance traveled of an open field (vehicle: 2120 ± 151 cm, *n* = 9; CORT: 2247 ± 116 cm, *n* = 8; *t*_(15)_ = 0.65, *p* = 0.52; Fig. 3*E*). Thus, decreased food-seeking was not because of altered locomotor activity. EPM test was used to test anxiety-like behavior. Seven day CORT mice exhibited decreased open arm time (vehicle: 103 ± 16 s, *n* = 9; CORT: 47 ± 13 s, *n* = 10; *t*_(17)_ = 2.7, *p* = 0.015; Fig. 3*F*) and open arm entries (vehicle: 29 ± 2, *n* = 9; CORT: 18 ± 4, *n* = 10; *t*_(17)_ = 2.5, *p* = 0.024; Fig. 3*G*), with no difference in distance traveled (vehicle: 21 ± 2, *n* = 9; CORT: 18 ± 3, *n* = 10; *t*_(17)_ = 0.81, *p* = 0.43; Fig. 3*H*). These results are consistent with anxiety-like behaviors in CORT mice, which could contribute to impaired reward-seeking behaviors.

**Figure 4.**
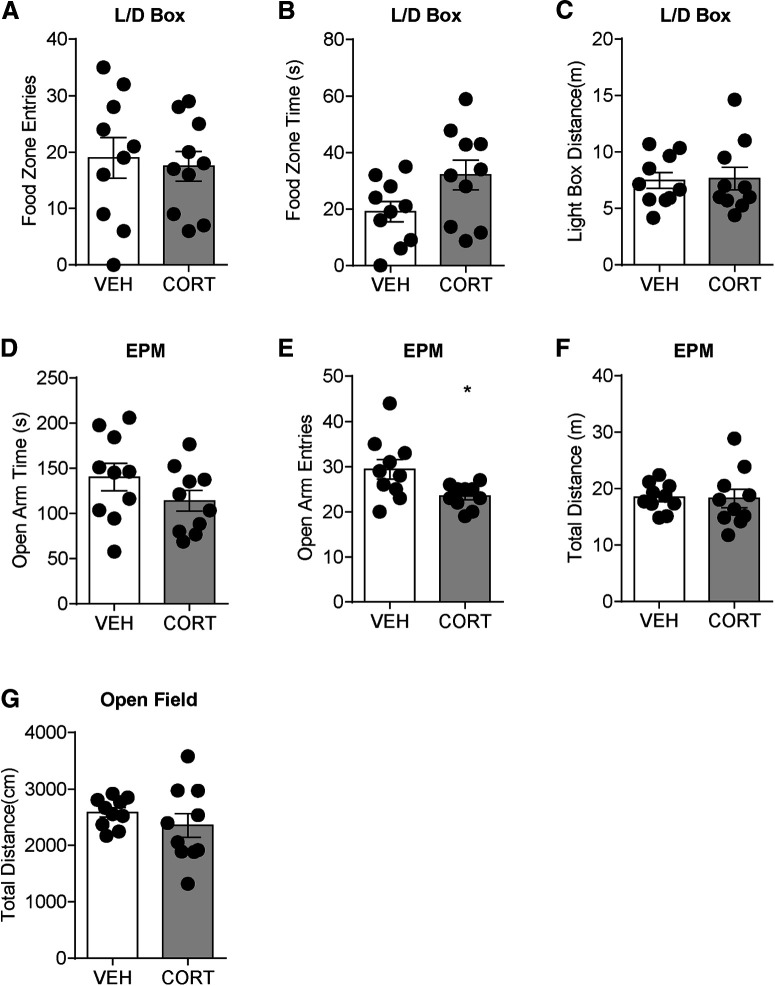
Reward-seeking behavior, anxiety-like behavior, and locomotor activity on 2 weeks after 7 d vehicle or CORT treatment. ***A***, Food zone entries. ***B***, Food zone time. ***C***, Light box distance in modified light/dark box test. ***D***, Open arm time. ***E***, Open arm entries. ***F***, Total distance in EPM. ***G***, Total distance in OF test. Error bars indicate SEM. *p < 0.05.

To test how long the 7 d CORT effects last, the same behavior paradigms were tested 2 weeks after CORT treatment in another group of mice. No significant change was found in food zone entries (vehicle: 19 ± 4, *n* = 10; CORT: 18 ± 3, *n* = 10; *t*_(18)_ = 0.34, *p* = 0.74; Fig. 4*A*), food zone time (vehicle: 19 ± 4 s, *n* = 10; CORT: 32 ± 5 s, *n* = 10; *t*_(18)_ = 2.0, *p* = 0.057; Fig. 4*B*), and light box distance traveled (vehicle: 8 ± 1 m, *n* = 10; CORT: 8 ± 1 m, *n* = 10; *t*_(18)_ = 0.14, *p* = 0.89; Fig. 4*C*) in the modified light-dark box test. Two weeks after 7 d CORT exposure, mice still displayed decreased open arm entries (vehicle: 29 ± 2, *n* = 10; CORT: 24 ± 1, *n* = 10; *t*_(18)_ = 2.5, *p* = 0.020; Fig. 4*E*) with no change in open arm time (vehicle: 140 ± 15 s, *n* = 10; CORT: 114 ± 12 s, *n* = 10; *t*_(18)_ = 1.4, *p* = 0.18; Fig. 4*D*) and distance traveled (vehicle: 18 ± 1 m, *n* = 10; CORT: 18 ± 2 m, *n* = 10; *t*_(18)_ = 0.12, *p* = 0.91; Fig. 4*F*) in the EPM test. There was no significant difference in total distance (vehicle: 2587 ± 82 cm, *n* = 10; CORT: 2352 ± 213 cm, *n* = 10; *t*_(18)_ = 1.0, *p* = 0.32; Fig. 4*G*) of the OF test.

### Decreased VTA dopamine neuron excitability and presynaptic glutamate release in 7 d CORT-treated mice

**Figure 5.**
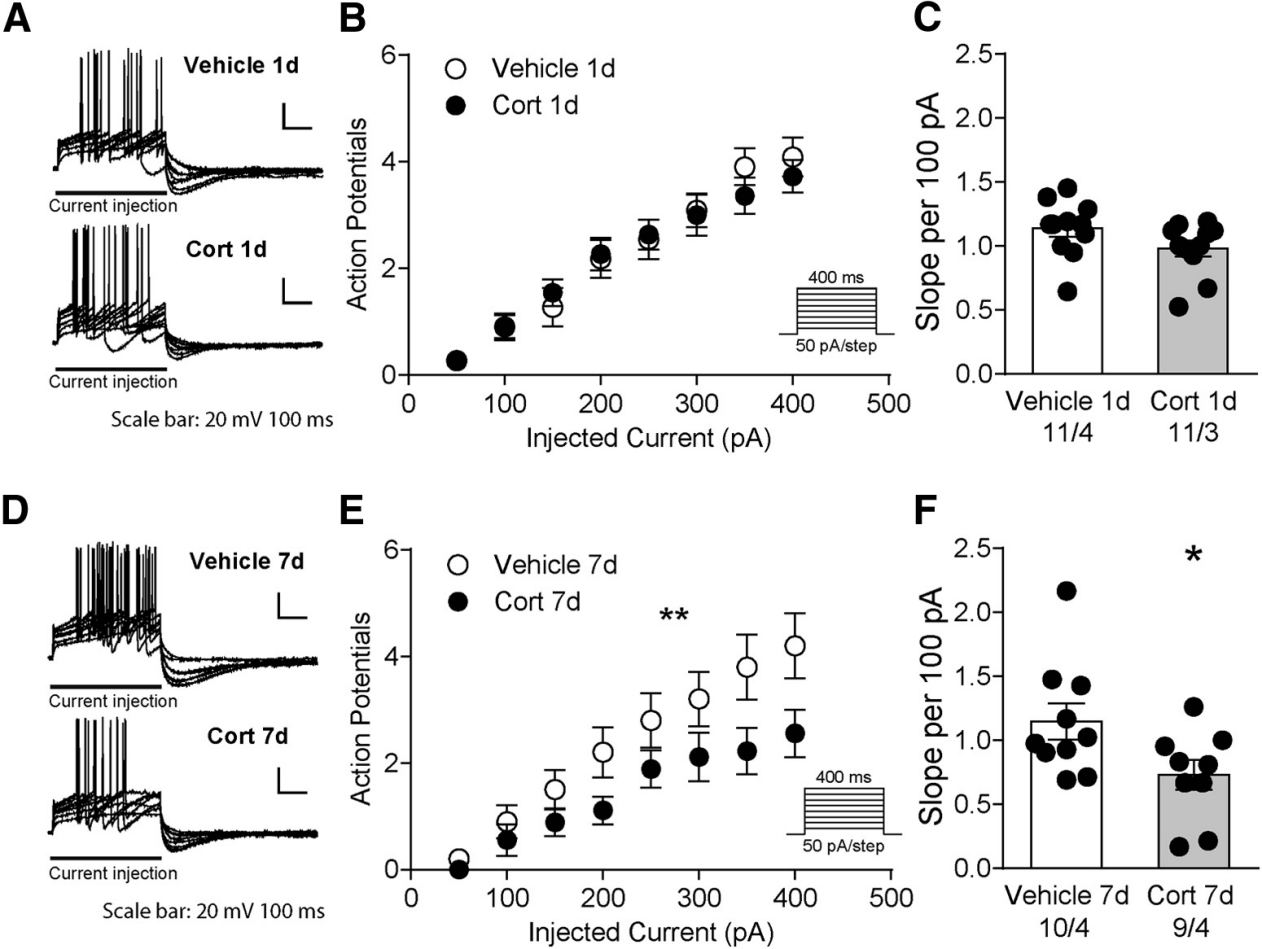
Decreased VTA dopamine neuron excitability in 7 d CORT mice. ***A***, Representative traces for neuron spikes elicited by current steps after 1 d treatment of vehicle or CORT. ***B***, The number of action potentials in response to current injection in 1 d treatment mice. ***C***, The slope of the number of spikes per 100 pA. ***D***, Representative traces for neuron spikes elicited by current steps after 7 d treatment of vehicle or CORT. ***E***, The number of action potentials in response to current injection in 7 d treatment mice. ***F***, The slope of the number of spikes per 100 pA in 7 d treatment mice. *p < 0.05. **p < 0.01.

To examine the excitability of VTA dopamine neurons after CORT treatment, we measured the spike number elicited by depolarizing current steps. One day CORT did not alter the current evoked firing or excitability of dopamine neurons compared with vehicle: (current injection × 1 d CORT interaction: *F*_(7,160)_ = 0.3825, *p* = 0.9116, Fig. 5*A*,*B*; slope: vehicle: 1.0 ± 0.06 per 100 pA (*N*/*n* = 11/3) vs 1 d CORT 1.1 ± 0.07 per 100 pA (*N*/*n* = 11/4), *t*_(20)_ = 1.7, *p* = 0.11, Fig. 5*C*). In contrast, dopamine neurons from 7 d CORT mice had reduced current-evoked firing and decreased excitability compared with vehicle treatment (current injection × 7 d CORT interaction: *F*_(7,119)_ = 3.147, *p* = 0.0044, Fig. 5*D*,*E*; slope: vehicle: 1.1 ± 0.1 per 100 pA (*N*/*n* = 10/4); 7 d CORT: 0.7 ± 0.1 per 100 pA (*N*/*n* = 9/4), *t*_(17)_ = 2.2, *p* = 0.038, Fig. 5*F*).

**Figure 6.**
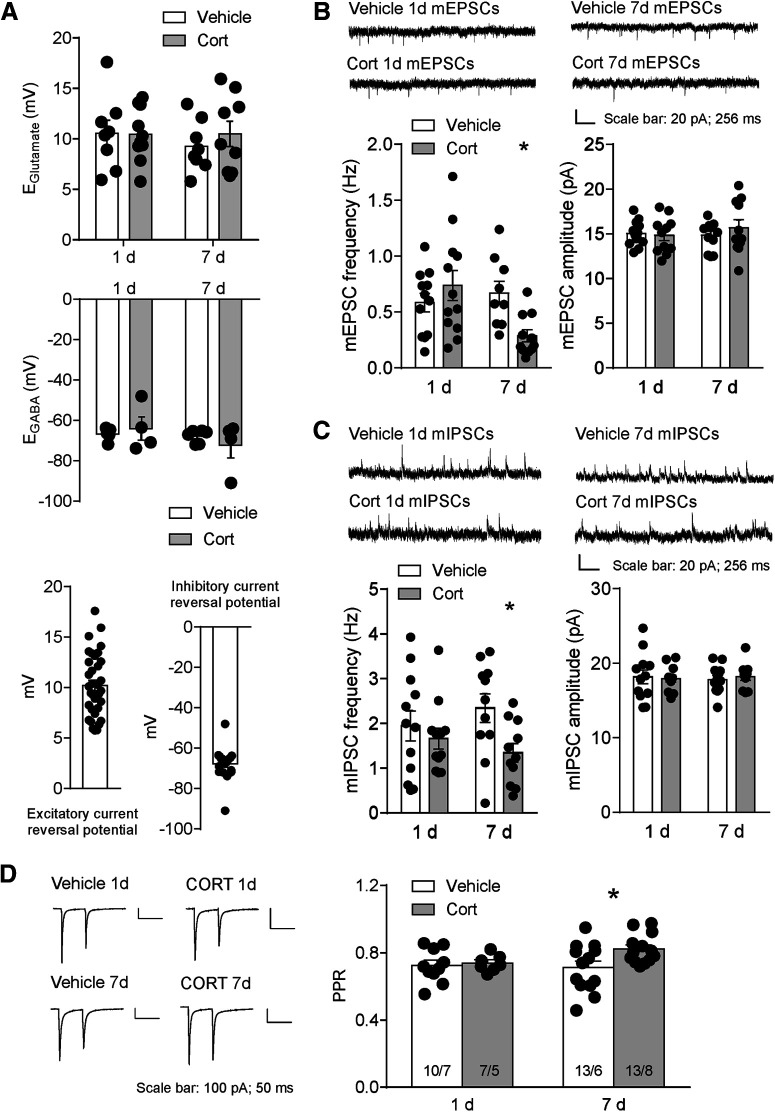
Decreased VTA dopamine neuron presynaptic glutamate release in 7 d CORT mice. ***A***, Excitatory reversal potential (top) and inhibitory reversal potential (middle) in vehicle and CORT mice on 1 and 7 d treatment, respectively. Data were pooled together to calculate excitatory current reversal potential and inhibitory current reversal potential (bottom). ***B***, Representative traces, frequency, and amplitude for mEPSCs in vehicle and CORT mice. ***C***, Representative traces, mIPSC frequency, and mIPSC amplitude for mIPSCs in vehicle and CORT mice. ***D***, Left, Representative traces for PPR. Right, PPR in 1 d and 7 d treatment mice. Error bars indicate SEM. *p < 0.05.

A shift in excitatory and inhibitory synaptic transmission may contribute to decreased dopamine neuronal excitability. To measure the effects of EPSCs and IPSCs on the same neuron, we tested inhibitory and excitatory current reversal potentials of dopamine neurons from vehicle mice and CORT-exposed mice, respectively. The EPSC reversal potential was not significantly different between vehicle mice and CORT-exposed mice, regardless of the duration of exposure (reversal potential × CORT interaction: *F*_(1,31)_ = 0.38, *p* = 0.54; Fig. 6*A*). Similarly, CORT treatment did not alter the IPSC reversal potential (reversal potential × CORT interaction: *F*_(1,15)_ = 0.92, *p* = 0.35; Fig. 6*A*). Pooled together, the average reversal potential for EPSCs was 10 ± 0.5 mV, *N*/*n* = 35/15, and for IPSCs was −68 ± 2 mV, *N*/*n* = 19/9; Fig. 6*A*). Using this information, we recorded mIPSCs at 10 mV and mEPSCs at −68 mV.

The mEPSC frequency was significantly decreased in 7 d CORT mice (vehicle: 0.6 ± 0.1 Hz, *N*/*n* = 11/4; CORT: 0.3 ± 0.06 Hz, *N*/*n* = 11/7; *t*_(20)_ = 2.3, *p* = 0.034), with no significant change in mEPSC amplitude (vehicle: 14.8 ± 0.6 pA; CORT: 15.7 ± 0.9 pA), suggesting decreased glutamate release probability onto the dopamine neurons in 7 d CORT mice (Fig. 6*B*). No significant change was observed in mEPSC frequency (vehicle: 0.6 ± 0.08 Hz, CORT: 0.7 ± 0.1 Hz, *N*/*n* = 12/5) or amplitude (vehicle: 15.1 ± 0.4 pA, CORT: 14.8 ± 0.5 pA, *N*/*n* = 12/5) in 1 d CORT mice (Fig. 6*B*). To further test the locus of synaptic effect, we measured the paired-pulse ratio (PPR), a measure that correlates with release probability (Branco and Staras, 2009). There was a significant increase in PPR after 7 d but not 1 d CORT (7 d CORT: vehicle: 0.7 ± 0.04, *N*/*n* = 13/6; CORT: 0.8 ± 0.02, *N*/*n* = 13/8; *t*_(24)_ = 2.4, *p* = 0.024; 1 d CORT: vehicle: 0.7 ± 0.03, *N*/*n* = 10/7; CORT: 0.7 ± 0.02, *N*/*n* = 7/5), suggesting a decrease in presynaptic release probability in 7 d CORT mice (Fig. 6*D*).

Similarly, mIPSC frequency was decreased in 7 d CORT mice (vehicle: 2.3 ± 0.3 Hz; CORT: 1.3 ± 0.2 Hz; *t*_(20)_ = 2.6, *p* = 0.017) with no significant change in the amplitude (vehicle: 17.8 ± 0.6 pA; CORT: 18.2 ± 0.5 pA) (Fig. 6*C*). Because both excitatory and inhibitory synaptic transmission was decreased in 7 d CORT mice, we did not see a significant change in excitatory/inhibitory (E/I) ratio (vehicle: 0.3 ± 0.04, *N*/*n* = 11/4; CORT: 0.2 ± 0.04, *N*/*n* = 11/7; *p* = 0.25). In 1 d CORT mice, mIPSC frequency (vehicle: 1.9 ± 0.3 Hz, CORT: 1.7 ± 0.2 Hz, *n* = 12/5), amplitude (vehicle: 18.2 ± 0.9 pA, CORT: 17.9 ± 0.5 pA, *N*/*n* = 12/5) (Fig. 6*C*), or E/I ratio (vehicle: 0.5 ± 0.1, CORT: 0.5 ± 0.1, *N*/*n* = 12/5) was not significantly different.

**Figure 7.**
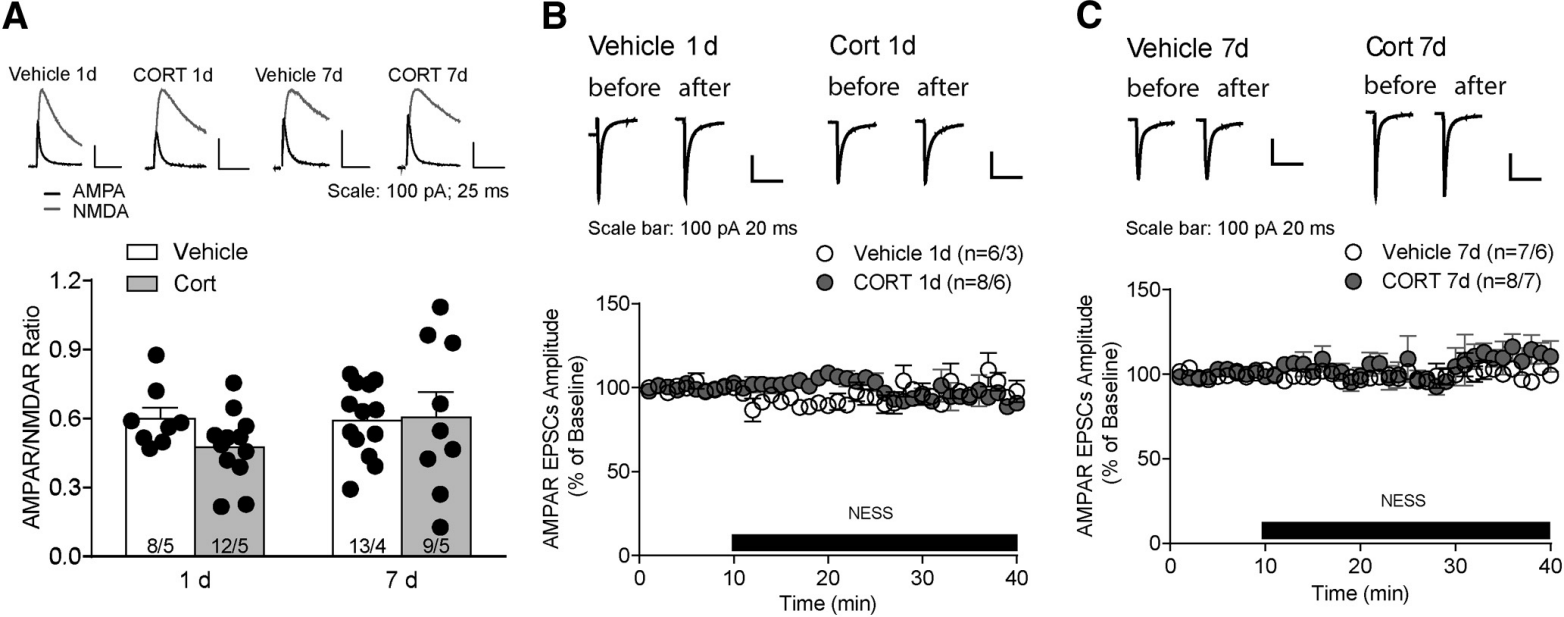
No change in AMPAR/NMDAR ratio and endocannabinoid tone in CORT mice. ***A***, Top, Representative traces for AMPAR/NMDAR ratio. Bottom, AMPAR/NMDAR ratio in vehicle and CORT mice. ***B***, Top, Representative traces for evoked EPSCs before and after the application of CB1R antagonist NESS in 1 d vehicle or CORT mice. Bottom, Evoked EPSC amplitude in the presence of CB1R antagonist. ***C***, Top, Representative traces for evoked EPSCs before and after the application of CB1R antagonist NESS in 7 d vehicle or CORT mice. Bottom, Evoked EPSC amplitude in the presence of CB1R antagonist. Error bars indicate SEM.

We measured the relative contribution of AMPAR and NMDAR EPSCs after vehicle or CORT treatment. The AMPAR/NMDAR ratio was not significant different between vehicle and CORT mice regardless of exposure duration (7 d CORT, vehicle: 0.6 ± 0.04, *N*/*n* = 13/4, CORT: 0.6 ± 0.1, *N*/*n* = 9/5, *t*_(20)_ = 0.15, *p* = 0.88; 1 d CORT, vehicle: 0.6 ± 0.05, *n* = 8/4, CORT: 0.5 ± 0.04, *n* = 12/5, *t*_(18)_ = 1.8, *p* = 0.08; Fig. 7*A*), suggesting there was no relative change in number or function of postsynaptic excitatory receptors.

Decreased presynaptic release probability may result from an increased endocannabinoid-mediated retrograde inhibition (Busquets-Garcia et al., 2018). In response to glucocorticoids, endocannabinoid induces a rapid suppression of synaptic transmission in the hypothalamus and the basolateral amygdala (Di et al., 2003; Di et al., 2016). To test whether CORT treatment alters endocannabinoid tone, evoked EPSCs were measured in the presence of a neutral CB1 receptor antagonist, NESS-0327. Application of NESS-0327 (0.5 μm) did not significantly change EPSC amplitude onto VTA neurons of vehicle or 1 d CORT mice (vehicle+NESS-0327: 93 ± 2% of baseline, *N*/*n* = 6/3; CORT+NESS-0327: 101 ± 4% of baseline, *N*/*n* = 8/6, *t*_(12)_ = 1.8, *p* = 0.10; Fig. 7*B*) or of vehicle or 7 d CORT mice (vehicle+NESS-0327: 99 ± 3% of baseline, *N*/*n* = 7/6; CORT+NESS-0327: 99 ± 5% of baseline, *N*/*n* = 8/7, *t*_(12)_ = 0.072, *p* = 0.94; Fig. 7*C*). Thus, decreased presynaptic release probability was not because of increased endocannabinoid tone.

### Chronic CORT treatment increased somatodendritic dopamine, and D2R antagonist sulpiride restores mEPSCs frequency and neuronal excitability

**Figure 8.**
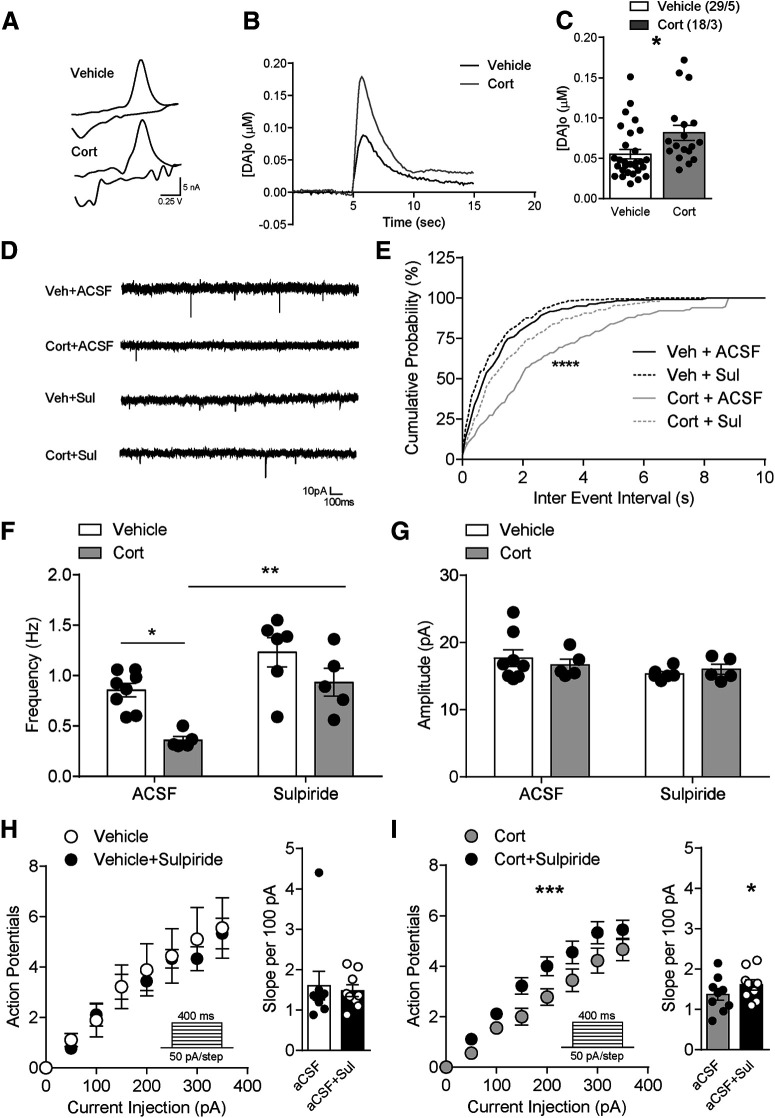
Increased VTA somatodendritic dopamine concentration in 7 d CORT mice, and D2R antagonist sulpiride restores mEPSCs frequency and neuronal excitability. ***A***, Example voltammograms from a single experiment for electrically evoked [DA]_o_ of vehicle or CORT-treated mice. ***B***, A representative current-time plot from a single experiment showing dopamine evoked in the VTA from a vehicle or CORT-treated animal. ***C***, Somatodendritic dopamine concentration in vehicle or CORT mice. ***D***, Example recordings of dopamine neuron mEPSCs, (***E***) cumulative probability plots for interevent interval, (***F***) mEPSC frequency, and (***G***) mEPSC amplitude from vehicle or CORT mice in the presence of either aCSF or sulpiride. ***H***, Neuronal excitability from vehicle mice in the presence of either aCSF or sulpiride. ***I***, Neuronal excitability from CORT mice in the presence of either aCSF or sulpiride. Error bars indicate SEM. *p < 0.05, **p < 0.01, ***p < 0.001, ****p < 0.0001

Somatodendritic dopamine regulates dopamine neuronal excitability through dopamine D2R-mediated autoinhibition (Jeziorski and White, 1989) and neurotransmission at presynaptic terminals expressing D2Rs (Pickel et al., 2002). To test whether 7 d CORT altered somatodendritic dopamine concentration [DA]_o_, we applied electrical stimulation (40 Hz, 20 pulses) to evoke dopamine release in VTA slices and measured the relative extracellular dopamine concentration ([DA]_o_) using fast-scan cyclic voltammetry (Fig. 8*A*,*B*). Evoked [DA]_o_ was significantly greater in 7 d CORT-treated compared with vehicle-treated mice (vehicle: 55 ± 6 nm, *N*/*n* = 29/5; CORT: 82 ± 9 nm, *N*/*n* = 18/3, Mann–Whitney test, *p* = 0.003; Fig. 8*B*,*C*), indicating that chronic CORT treatment increases somatodendritic dopamine.

We next examined whether decreased neuronal transmission and excitability resulted from enhanced D2R signaling by elevated somatodendritic dopamine. Bath application of the D2R antagonist sulpiride (5 μm) restored mEPSC interevent interval cumulative probability (Fig. 8*E*; Kolmogorov–Smirnov test) and mEPSCs frequency ((Fig. 8*F*) in VTA neurons from CORT mice (main effect of CORT: *F*_(1,20)_ = 20.15, *p* = 0.0002 and sulpiride: *F*_(1,20)_ = 14.14, *p* = 0.0012; vehicle+aCSF: 0.9 ± 0.07 Hz, *N*/*n* = 8/5; CORT+aCSF: 0.4 ± 0.04 Hz, *N*/*n* = 5/4; vehicle+sulpiride: 1.2 ± 0.2 Hz, *N*/*n* = 6/6; CORT+sulpiride: 0.9 ± 0.1 Hz, *N*/*n* = 5/3; Fig. 8*D–F*). There was no significant interaction between CORT and sulpiride treatment on mEPSC amplitude (vehicle+aCSF: 18 ± 1 pA, *N*/*n* = 8/5; CORT+aCSF: 17 ± 0.9 pA, *N*/*n* = 5/4; vehicle+sulpiride: 15 ± 0.3 pA, *N*/*n* = 6/6; CORT+sulpiride: 16 ± 0.7 pA, *N*/*n* = 5/3; *F*_(1,20)_ = 0.68, *p* = 0.42) (Fig. 8*G*). Together, these data indicate that D2Rs are at least partially required for decreased release probability of glutamate on to VTA dopamine neurons of 7 d CORT-treated mice.

To test the impact of D2R signaling blockade on neuronal excitability, we measured the spike number elicited by depolarizing current steps with or without application of sulpiride. Sulpiride did not change firing (*t*_(8)_ = 0.4, *p* = 0.68) or slope of the frequency-current plot, suggesting that, in vehicle-treated mice, there is no dopaminergic tone acting at D2R receptors (Fig. 8*H*). In contrast, sulpiride significantly increased the excitability (CORT × sulpiride interaction: repeated-measures ANOVA: *F*_(7,56)_ = 4.8, *p* = 0.0003) and frequency-current slope of VTA neurons from 7 d CORT mice (slope: 1.4 ± 0.2 per 100 pA, *N*/*n* = 9/4-1.6 ± 0.1 per 100 pA, *N*/*n* = 9/4, *t*_(8)_ = 2.6, *p* = 0.031) (Fig. 8*I*).

### D2R signaling blockade in the VTA restores food-seeking behaviors and alleviates anxiety-like behavior, but does not rescue motivated behavior

**Figure 9.**
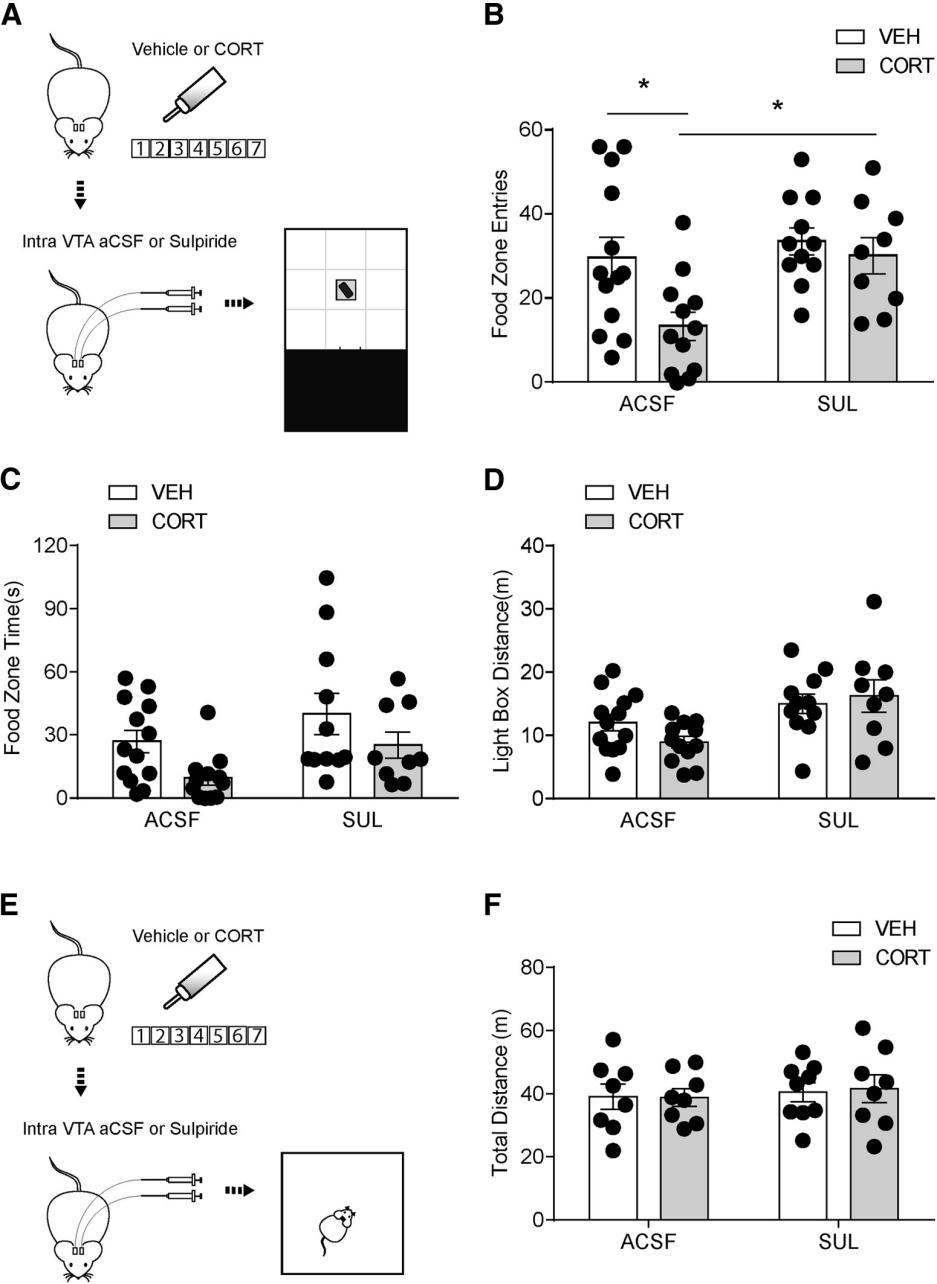
Decreased reward-seeking behavior can be reversed by intra-VTA sulpiride infusion with no change in locomotor activity. ***A***, Flowchart for reward-seeking behavior test, (***B***) food zone entries, (***C***) food zone time, and (***D***) distance traveled in the light box in vehicle and CORT mice with either intra-VTA aCSF or sulpiride infusion. ***E***, Flowchart for OF test. ***F***, Total distance traveled in the open field with either intra-VTA aCSF or sulpiride infusion. Error bars indicate SEM. *p < 0.05.

To investigate the effect of a D2R antagonist on food-seeking behaviors, we infused with either aCSF or sulpiride intra-VTA before the modified light-dark box test (Fig. 9*A*). Decreased food zone entries in 7 d CORT mice (vehicle+aCSF: 30 ± 5, *n* = 13; CORT+aCSF: 13 ± 3, *n* = 12) were restored by intra-VTA sulpiride with a main effect of CORT treatment (*F*_(1,41)_ = 5.69, *p* = 0.016) and sulpiride infusion (*F*_(1,41)_ = 6.28, *p* = 0.022; Fig. 9*B*). There was no interaction between CORT treatment and drug treatment on food zone time (*F*_(1,41)_ = 0.04, *p* = 0.85, vehicle+aCSF: 27 ± 5 s, *n* = 13; CORT+aCSF: 10 ± 3 s, *n* = 12; vehicle+sulpiride: 40 ± 10 s, *n* = 11; CORT+sulpiride: 25 ± 6 s, *n* = 9; Fig. 9*C*), with main effects of CORT (*F*_(1,41)_ = 6.11, *p* = 0.02) and intra-VTA sulpiride (*F*_(1,41)_ = 4.8, *p* = 0.03). Vehicle and 7 d CORT mice treated with either aCSF or sulpiride had no significant difference in distance traveled in the light box (vehicle+aCSF: 12 ± 1 m, *n* = 13; CORT+aCSF: 9 ± 0.9 m, *n* = 12; vehicle+sulpiride: 15 ± 2 m, *n* = 11; CORT+sulpiride: 16 ± 3 m, *n* = 9, main effect of CORT: *F*_(1,41)_ = 0.35, *p* = 0.6; Fig. 9*D*). In addition, sulpiride did not change total distance traveled in the OF test (vehicle+aCSF: 39 ± 4 m, *n* = 8; CORT+aCSF: 39 ± 3 m, *n* = 8; vehicle+sulpiride: 41 ± 3 m, *n* = 9; CORT+sulpiride: 42 ± 4 m, *n* = 8, *F*_(1,29)_ = 0.032, *p* = 0.86, main effect of CORT: *F*_(1,29)_ = 0.012, *p* = 0.9; Fig. 9*E*,*F*). These data indicate that D2R inhibition can restore food-seeking with no change in locomotor activity in 7 d CORT mice.

**Figure 10.**
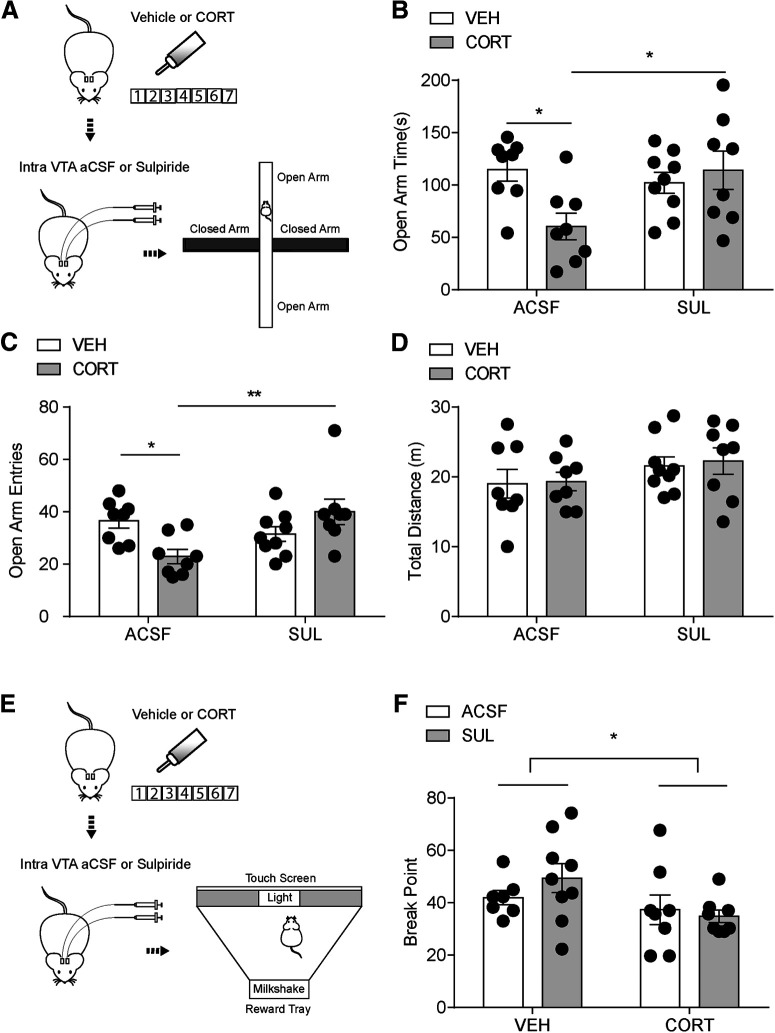
Intra-VTA sulpiride infusion alleviates anxiety-like behavior but does not rescue motivated behavior. ***A***, Flowchart for EPM test, (***B***) open arm time, (***C***) open arm entries, and (***D***) total distance for vehicle and CORT mice with either intra-VTA aCSF or sulpiride infusion. ***E***, Flowchart for PR test. ***F***, Breakpoint for vehicle and CORT mice with either intra-VTA aCSF or sulpiride infusion. Error bars indicate SEM. *p < 0.05. **p < 0.01.

To further test whether the restoration of food-seeking behaviors is because of alleviated anxiety-like behavior or normalized motivated behavior, we tested vehicle and CORT mice in the EPM test (Fig. 10*A*) and operant chamber (Fig. 10*E*) with intra-VTA aCSF or sulpiride infusion, respectively. Sulpiride restored CORT-induced reductions in open arm time (vehicle+aCSF: 115 ± 11 s, *n* = 8; CORT+aCSF: 60 ± 13 s, *n* = 8; vehicle+sulpiride: 102 ± 10 s, *n* = 9; CORT+sulpiride: 114 ± 18 s, *n* = 8, *F*_(1,29)_ = 6.28, *p* = 0.018; Fig. 10*B*) and open arm entries (vehicle+aCSF: 37 ± 3, *n* = 8; CORT+aCSF: 23 ± 3, *n* = 8; vehicle+sulpiride: 31 ± 3, *n* = 9; CORT+sulpiride: 40 ± 5, *n* = 8, *F*_(1,29)_ = 10.88, *p* = 0.0026; Fig. 10*C*), with no effects on total distance (vehicle+aCSF: 19 ± 2 m, *n* = 8; CORT+aCSF: 19 ± 1 m, *n* = 8; vehicle+sulpiride: 22 ± 1 m, *n* = 9; CORT+sulpiride: 22 ± 2 m, *n* = 8, *F*_(1,29)_ = 0.015, *p* = 0.90; Fig. 10*D*) in the EPM test. PR schedule was used in operant chamber test to assess motivated behavior. Breakpoint was significantly less in CORT mice (vehicle+aCSF: 42 ± 3, *n* = 7; CORT+aCSF: 37 ± 6, *n* = 8; vehicle+sulpiride: 49 ± 5, *n* = 9; CORT+sulpiride: 35 ± 2, *n* = 8, CORT main effect, *F*_(1,28)_ = 4.45, *p* = 0.044; Fig. 10*F*). However, sulpiride had no main effect on instrumental performance (*F*_(1,28)_ = 0.30, *p* = 0.59), and no interaction between CORT and sulpiride was observed (*F*_(1,28)_ = 1.20, *p* = 0.28). These results suggest that D2R signaling blockade in the VTA of CORT mice can alleviate anxiety-like behavior but does not rescue motivated behavior.

## Discussion

**Figure 11.**
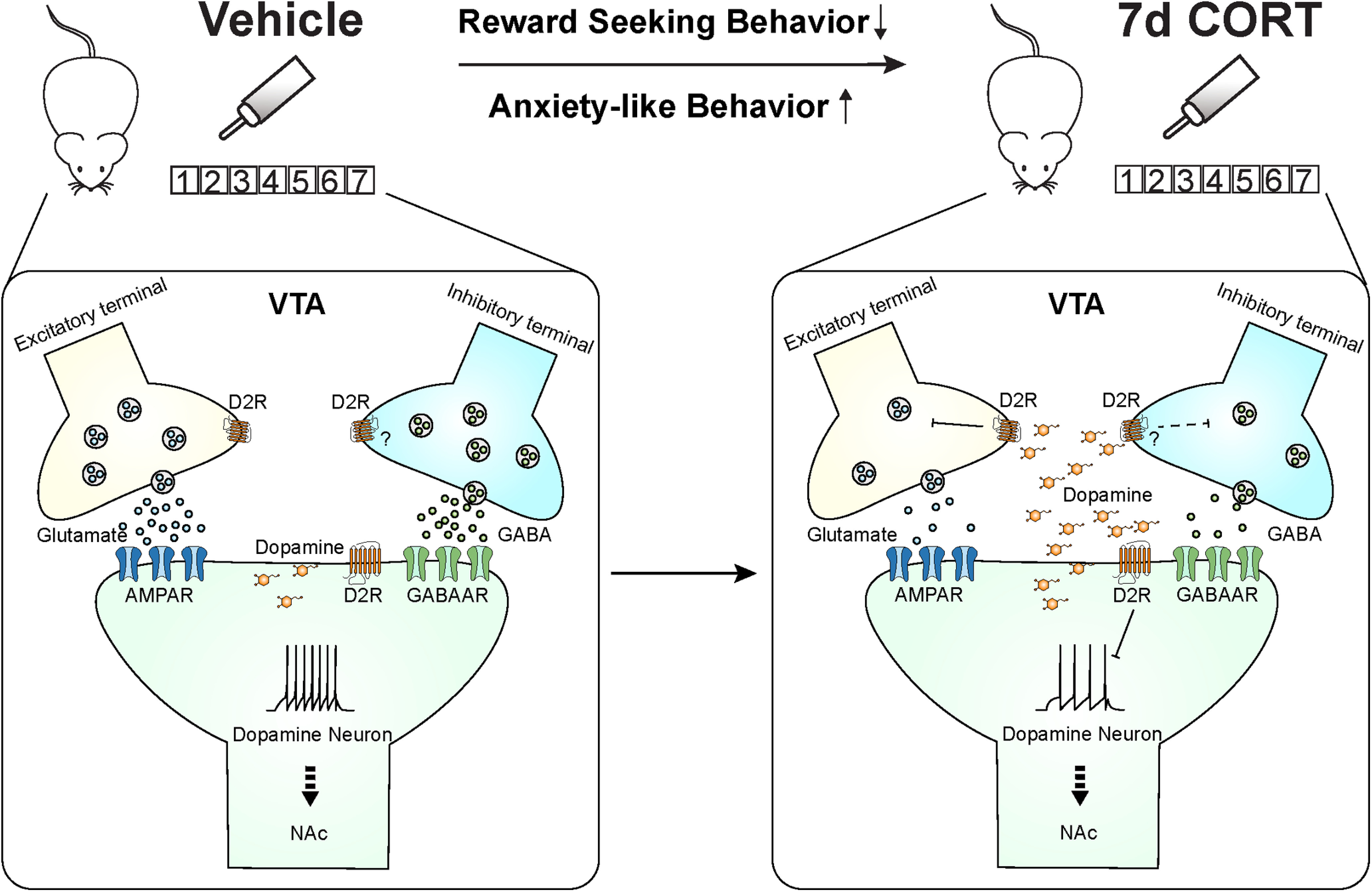
Schematic diagram showing mechanism underlying 7 d CORT treatment impairment of food-seeking behavior and anxiety-like behavior. A critical node implicated in this dysfunction is the VTA. Decreased excitability and synaptic transmission onto the dopamine neurons in CORT mice are likely mediated by increased somatodendritic dopamine activating presynaptic and postsynaptic D2R signaling.

Here, we report chronic CORT treatment impairs food-seeking behavior and elevates anxiety-like behavior in mice. Furthermore, we observed decreased excitability and synaptic transmission onto dopamine neurons of chronic CORT-treated mice. This was likely mediated by increased somatodendritic dopamine-activating presynaptic and postsynaptic D2Rs (schematic, Fig. 11). Blockade of D2Rs restored food-seeking behavior in a mildly aversive environment and alleviated anxiety-like behavior in chronic CORT-exposed mice. Together, CORT-induced neuronal disruptions in a key region mediating reward-seeking and may underlie CORT-induced anhedonia and neuropsychiatric sequelae.

### CORT induced altered reward-seeking behavior, elevated anxiety-like behavior and metabolic dysfunction in mice

While sated vehicle-treated mice will explore and engage with palatable food in the light box, consistent with previous work establishing this model of nonhomeostatic feeding (Teegarden and Bale, 2007; Cottone et al., 2012; Liu et al., 2016), mice treated with 7 d CORT have reduced food-seeking in this paradigm. It is likely that enhanced anxiety-like behavior contributed to reduced food approach behavior, given that, in this food-seeking model, mice must overcome their innate aversion to light open spaces. Alternatively, this may be because of increased satiety in CORT-treated mice as they consume more home cage food. While others have demonstrated that prolonged CORT treatment (4 weeks) reduces home cage locomotor activity (Karatsoreos et al., 2010), 7 d CORT-treated mice do not exhibit locomotor impairment. Regardless, these results suggest that 7 d CORT treatment induces an altered motivational state to reduce food-seeking. Indeed, exogenous CORT administration in humans reveals a downregulation of the limbic reward system, and thereby diminishes reward anticipation and motivational processing (Tidey and Miczek, 1996; Berton et al., 2006).

Cushing's syndrome is associated with elevated CORT, which presents in males at a younger age with more significant CORT elevation and more severe clinical symptoms (Kleen et al., 2006). Chronic oral CORT treatment in mice has been characterized as a model of Cushing's syndrome (Kinlein et al., 2017). Prolonged CORT treatment initiated at postnatal day 35 leads to an initial drop in weight during the first week of treatment followed by significant weight gain (Karatsoreos et al., 2010). This is associated with increased adiposity as well as impaired glucose tolerance (Karatsoreos et al., 2010; Kinlein et al., 2017). Consistent with these studies, our results show that 7 d CORT treatment in periadolescent male mice have decreased weight gain, lower basal glucose level, hyperphagia, and increased water intake.

### VTA neuronal excitability and synaptic transmission in response to CORT

The excitability of VTA dopamine neurons plays a crucial role in different motivational states, as a switch from tonic to phasic firing can enhance behavioral activation (Tsai et al., 2009; Zweifel et al., 2009). Previous studies indicate that acute CORT treatment can influence the mesolimbic dopamine system. Acute CORT injection decreases dopamine clearance and elevates dopamine concentration in the NAc (Graf et al., 2013; Wheeler et al., 2017). The VTA is a major source of dopamine inputs to NAc, and the activity of the VTA dopamine neurons impacts dopamine release in the NAc. We found that chronic CORT treatment markedly decreased excitability of VTA dopamine neurons. CORT may change neuronal excitability by directly targeting GR- or MR-expressing dopamine neurons to influence cellular activity or indirectly acting on upstream brain regions targeting VTA neurons. After chronic CORT treatment, hypothalamic GR-expressing CRH neurons exhibit reduced excitability (Bittar et al., 2019), whereas hippocampal neurons have enhanced excitatory postsynaptic receptor responses (Karst and Joels, 2005). Activation of these projections could drive VTA dopamine release. Because chronic CORT treatment increased somatodendritic dopamine, it is possible that enhanced dopamine targets D2R to suppress VTA dopamine firing rate (Jeziorski and White, 1989). Indeed, inhibition of D2R with sulpiride in CORT-treated mice restored the excitability of dopamine neurons. Notably, D2Rs are expressed on extrasynaptic dendrites of VTA dopamine neurons near excitatory synapses (Pickel et al., 2002); therefore, D2R activation via somatodendritic dopamine is likely to dampen the influence of excitatory inputs.

Dopamine neurons in the VTA innervate brain regions that are critical for emotional processing (Russo and Nestler, 2013) and mediate symptoms of anxiety, including NAc, amygdala, PFC, and hippocampus. Anxiety-like responses, such as reduced open arm time in the EPM test and decreased head-dipping in the hole-board test, can be modulated by dopaminergic receptors in the amygdala (Bananej et al., 2012), hippocampus (Zarrindast et al., 2010; Nasehi et al., 2011), and PFC (Broersen et al., 2000; Wall et al., 2004). Impaired dopamine neuronal activation can result in enhanced anxiety-like behaviors (Zweifel et al., 2011). Therefore, impaired dopamine neuron excitability in CORT mice can contribute to an elevated anxiety-like phenotype via a variety of corticolimbic circuits.

Systemic dexamethasone or forced swim test increases the AMPAR/NMDAR ratio of VTA dopamine neurons, an effect blocked by the GR/progesterone receptor inhibitor, RU486 (Saal et al., 2003). However, while 1 d CORT self-administration was not sufficient to change synaptic transmission, chronic CORT treatment reduced both excitatory and inhibitory synaptic transmission. Because there was no shift in the E/I balance onto dopamine neurons of CORT-treated mice, the decrease in firing rate is unlikely because of an altered synaptic input, but rather an intrinsic change to dopamine neurons. Decreased mEPSC frequency, but not amplitude, and a paired-pulse facilitation of glutamate release onto dopamine neurons was consistent with a change in presynaptic release probability. However, this effect was not because of elevated endocannabinoid tone. By contrast, D2R inhibition reversed the 7 d CORT-induced suppression of mEPSC frequency, suggesting that CORT-induced elevated somatodendritic dopamine could act at D2Rs to suppress release probability. The presynaptic inhibition could be general or specific to some afferents depending on the expression of D2R. D2Rs are primarily expressed on dopamine neuronal dendrites and somata but have also been localized to nondopaminergic axon fibers in the VTA as well as astrocytes (Sesack et al., 1994). The restoration of synaptic transmission by D2R inhibition rules out possible synaptic scaling from CORT exposure as has been observed in hypothalamic CRH neurons (Bittar et al., 2019). Together, chronic CORT increases somatodendritic dopamine, which acts at D2R to suppress excitatory synaptic transmission onto dopamine neurons. Although there is no direct evidence for D2R expression on inhibitory terminals in the VTA, one study suggests that activation of D2R at inhibitory terminals facilitates endocannabinoid-mediated long-term depression in the VTA DA neurons (Pan et al., 2008). Further study should test the mechanisms of how CORT treatment suppresses inhibitory synapses and determine the D2R-sensitive excitatory inputs suppressed with chronic CORT exposure.

Inhibition of D2Rs in the VTA also restored the CORT-induced suppression of food-seeking, consistent with previous studies showing VTA D2R availability is inversely associated with incentive motivation (de Jong et al., 2015) and novelty seeking (Zald et al., 2008; Tournier et al., 2013). Previous reports have indicated that systemic administration of a high (50 mg/kg) but not low (25 mg/kg) dose of sulpiride decreases rats' responding under a PR schedule of reinforcement (Heath et al., 2015; Sakamoto et al., 2015). Notably, we did not observe a significant effect of intra-VTA sulpiride infusion on breakpoint in control mice. Consistent with this, mice lacking D2Rs show similar acquisition in self-administration for natural reward compared with WT mice (Holroyd et al., 2015). Because VTA D2R blockade alleviated anxiety-like behavior but did not rescue instrumental performance, the antianxiety effect of D2R blockade likely contributes to restored reward-seeking behavior. Further study needs to tackle which pathway is involved in the CORT-induced suppression of motivated behavior.

CORT is a mediator of stress responses and provides feedback to the CNS that controls cognitive and emotional processing. Although CORT administration through the drinking water does not fully represent a physiological stress response, it can explicitly manipulate CORT concentration and recapitulate chronic stress in critical aspects of the neuroendocrine response, justifying it a valuable approach for understanding the stress-related pathology. Acute stress modulates dopamine neurons in a projection-specific way. Although the effects are complex, one prominent observation is increased dopamine release in the NAc and PFC in response to an acute stressor (Tidey and Miczek, 1996; Young, 2004; Lammel et al., 2011). Chronic stress induced mood and motivation deficits can be attributed to dysfunction of mesolimbic dopamine pathways (Berton et al., 2006; Mehta et al., 2010). Consistent with our study showing decreased DA neuronal excitability in chronic CORT mice, long-term psychological stress in humans dampens striatal dopaminergic function (Bloomfield et al., 2019). Therefore, D2 autoinhibition of DA release can be tested as a potential underlying mechanism in chronic stress-induced deficits.

In conclusion, chronic CORT decreases neuronal excitability in the VTA and food-seeking behavior in a mildly aversive environment. This effect requires activation of D2Rs likely through elevated somatodendritic dopamine. Although food approach behaviors are largely restored by blocking D2R signaling in the current paradigm, the full recovery of reward-seeking behavior may also require involvement of other brain regions that may have distinct mechanisms influenced by chronic CORT. These results suggest that neuropsychiatric side effects of CORT medication related to reward dysfunction may be ameliorated with D2R antagonists.
